# A Recent Remedy for Phthisis

**Published:** 1888-03

**Authors:** 


A Recent Remedy for Phthisis.—It having been observed that those exposed, in the manufacture of hydrofluoric acid (H. F.), to its fumes, were greatly relieved of phthisical symptoms, Garcin began to experiment with this acid. In a year, he has treated 100 tuberculous patients, of which thirty-five were cured, forty-one benefited, and only ten died. The method of treatment is simple. The patient is placed each day in a room containing six cubic metres of air saturated with hydrofluoric gas obtained by passing a current of air, by means of a pump, through a bottle containing 300 grams of distilled water, and 100 grams of hydro-fluoric acid. For a patient in early phthisis, twenty litres of gas to a cubic metre is employed; but in advanced cases, only ten litres should be used.—*Rev. Sc.*

